# Early Life Nutrition: The First 1000 Days and Healthy Aging in Cystic Fibrosis

**DOI:** 10.3390/nu18050739

**Published:** 2026-02-25

**Authors:** Caitlin N. Miles, Gillian M. Nixon, Zoe E. Davidson

**Affiliations:** 1 Department of Nutrition, Dietetics and Food, Monash University, Melbourne, VIC 3168, Australia; 2 Nutrition and Dietetics, Monash Children’s Hospital, Melbourne, VIC 3168, Australia; 3 Respiratory Medicine, Monash Children’s Hospital, Melbourne, VIC 3168, Australia; 4 Department of Paediatrics, Monash University, Melbourne, VIC 3168, Australia

**Keywords:** cystic fibrosis, child, infant, nutrition, breastfeeding, diet, growth and development

## Abstract

Achievement of optimal nutritional status within the first 1000 days of life for a child with cystic fibrosis (CF) is of paramount importance, with an emphasis on favorable early life growth trajectories that best optimize pulmonary and extrapulmonary health. The ‘first 1000 days’ framework emphasizes that environmental, sociocultural and nutritional exposures during this period can have life-long consequences for physical, cognitive, social and emotional health and development. Optimal nutrition encompasses not just physical growth, but the provision of nutrients and optimal feeding throughout the preconception, pregnancy and first 1000-day period to ensure lifelong healthy development and aging. For children with CF (cwCF), the first 1000 days is marred by a myriad of complications, exposing a unique nutritional fragility within this critical developmental window. Conversely, as life expectancy increases for people with CF (pwCF), overnutrition is becoming increasingly prevalent and the widespread uptake of disease-modifying drugs challenges clinicians to take a nuanced and personalized approach to lifelong nutritional care. This review explores early disease manifestations of CF and their impact on early life growth and nutrition in the modern era of CF. This review also considers how we might theoretically view early life nutrition in CF from a lens which takes into consideration well-known frameworks such as ‘the first 1000 days’ and ‘developmental origins of health and disease’.

## 1. Introduction

Cystic fibrosis (CF) is an autosomal recessive genetic disorder affecting more than 160,000 people worldwide [1]. Once a life-limiting disease, CF is now broadly considered a ‘disease for life’ due to major advances in therapeutic care, notably the emergence of disease-modifying drugs. Children with CF (cwCF) are not only growing into adults with CF, but children born today are likely to live long enough to require targeted geriatric care [2,3]. Non-communicable diseases, previously uncommon in people with CF (pwCF), are now being documented at higher rates than the general population, including metabolic syndrome, cardiovascular disease (CVD) and cancer [3,4,5]. Overweight and obesity is now a concern for 22–43% of people living with CF [3,6,7].

Nutrition has been a key pillar of CF care since the disease was first pathologically described in the early 20th century [8], with optimal nutritional status strongly predictive of lung health and survival [9,10,11,12]. Nutritional interventions can be aggressive in the early months and years, with the primary goal to correct growth deficits, manage symptoms of exocrine pancreatic insufficiency (EPI) and limit early life lung disease. As a result of early disease manifestations, optimal infant nutrition and feeding development may be compromised and the early feeding journey marked with challenges [13,14,15,16].

There is an abundance of literature in the general population suggesting that physical and psychological development, health and wellbeing and later life chronic disease are associated with nutritional practices and behaviors seen during the first two years of life [17,18,19,20,21,22,23,24]; a period of time often referred to as ‘the first 1000 days’ [25,26]. Nutritional practices, such as prenatal nutrition, breastfeeding and the timely introduction of nutrient dense complementary foods, are known to shape a child’s future health and the avoidance or development of later life chronic disease [25]. Frameworks such as ‘the first 1000 days’ and ‘developmental origins of health and disease’ seek to explain these phenomena.

With early life commencement of disease-modifying drugs, alongside continued therapeutic advances in care and extended life span, it is of utmost importance that CF nutritional practices in infancy and early childhood are optimized to ensure developmental and health outcomes are comparable to that of children without CF. The aim of this narrative review is to summarize the literature on early life nutrition in CF with a focus on the first 1000 days of life. The impact of early CF disease expression on the attainment of optimal nutrition will be explored, alongside the pillars of prenatal nutrition, breastfeeding and nutrient dense complementary feeding. We hypothesize how these may influence developmental outcomes and lifelong healthy aging in the CF population. Any known or emerging influences of disease-modifying drugs on early life nutrition within the first 1000 days of life will be encompassed in this review.

## 2. Methodology

### 2.1. Reporting Checklist

The ‘Scale for the Assessment of Narrative Review Articles’ (SANRA) checklist [27] has been utilized in the preparation of this narrative review to ensure quality, transparency and consistency within the narrative review field.

### 2.2. Literature Search

MEDLINE (Ovid) and Google Scholar were utilized as key information sources for the preparation of this review. Studies were limited to full-text English-language studies; unpublished material including conference abstracts were excluded; however, grey literature produced by government or non-profit organizations, such as the World Health Organization (WHO) or the CF Foundation, were included. Literature searches were performed between October 2025 and January 2026 and there were no limitations placed on date. The Population-Concept-Context framework was used to design the search. Population was largely defined as an infant or cwCF, of any age, but also included adults with CF when there was insufficient evidence in the pediatric CF population, or any infant or child when referring to non-CF populations. Concepts were broad but included terms such as ‘the first 1000 days’, ‘prenatal nutrition’, ‘breastfeeding’, ‘infant formula’, ‘complementary solids’, ‘development’, ‘chronic disease’ and ‘developmental origins of health and disease’. Contexts included key terms such as ‘CFTR modulator’ with key time periods such as pre-CFTR modulator therapy (i.e., prior to 2012) and post-CFTR modulator therapy (i.e., post 2012). There were no limits or specifications on country or setting. A systematic quality appraisal of individual studies was not conducted; however, the principles of bias, validity, precision and applicability were considered by authors when assessing evidence for inclusion in this review.

### 2.3. Application of the ‘First 1000 Days’ and ‘Developmental Origins of Health and Disease’ Concepts

The ‘first 1000 days’ concept, introduced over three key *Lancet* series published in 2007, 2011 and 2017, highlights a significant body of evidence linking child developmental potential with key environmental, sociocultural and nutritional exposures occurring between conception and two years of age [28,29,30,31]. The framework was originally developed to focus on the millions of children living in low- and middle-income countries (LMICs) who were not meeting their developmental potential due to malnutrition and poverty and encompassed a broad umbrella of ‘nurturing care’ requirements: health, nutrition, security and safety, responsive caregiving and early learning [32]. Since these hallmark series, the ‘first 1000 days’ framework, or components of, have been applied across other vulnerable populations, including premature infants [33,34,35], low socioeconomic communities [36,37] and indigenous Australian children [38,39]. This narrative review will focus on the nutritional pillars of the framework and how these can be theoretically applied to the CF context: prenatal nutrition, breastfeeding and nutrient-dense complementary foods.

The ‘developmental origins of health and disease’ (DOHaD) framework is based on an abundance of literature demonstrating that early life environmental exposures, including nutrition, during critical windows of fetal and neonatal development, permanently program physiological and metabolic processes, influencing the development of later life cardiometabolic and noncommunicable diseases [24]. Also referred to as ‘fetal origins of adult disease’, the framework has evolved to include the body of evidence linking exposures during both prenatal and postnatal developmental windows. Longitudinal birth cohort studies from both developed and developing countries suggest that malnutrition in the first 1000 days of life, followed by accelerated growth in childhood and adolescence, predisposes children to an increased risk of cardiometabolic conditions [19,20,22,40,41,42,43]. DOHaD suggests that fetuses and infants adapt to limited nutrition by programming changes in their genetic expression, physiology and metabolism [24,44,45]. Then, when exposed to an abundance of nutrition later in childhood, these early metabolic and physiological adaptations, such as the ability to conserve energy and store fat efficiently, may become maladaptive, ultimately leading to an elevated risk of cardiometabolic and noncommunicable diseases as adults. Again, for the purpose of this review, only the nutritional elements of the DOHaD framework will be theoretically applied to the CF context. It is the intention of this review to elicit thought and discussion and to generate hypotheses within this rapidly changing field.

## 3. Cystic Fibrosis: An Evolving Multisystem Disease

### 3.1. Pathophysiology

CF is a genetic disorder resulting in defects in the cystic fibrosis transmembrane conductance regulator (CFTR) protein, a chloride ion channel, which primarily acts to regulate the transport of chloride ions across apical membranes of epithelial cells [46]. When abnormalities in the production, transport or function of the CFTR protein occur, fluid and electrolyte balance is disrupted. This disruption causes a cascade of physiological changes in the affected organs: epithelial cell surfaces become dehydrated with an accumulation of viscid mucus, bacteria proliferate in the lungs, ducts are blocked and widespread inflammatory and immune responses escalate. Ultimately, organ damage occurs to the lungs, pancreas, hepatobiliary system, gastrointestinal and reproductive tracts [46].

### 3.2. Diagnosis

The diagnosis of CF is a multistep process involving clinical history, sweat chloride test and gene mutation analysis [3,7]. The majority of infants with CF in countries such as the United States (US), Canada, the United Kingdom (UK) and Australia are initially identified via newborn screening, followed by a confirmatory gene mutation diagnosis and/or sweat chloride test [47,48]. Newborn screening and the early detection of CF allows for early intervention to prevent or minimize progressive and irreversible lung damage and to optimize nutrition. Newborn screening has contributed to the improved health outcomes and exponential survival curve seen in the last several decades of CF care [48]. The next most common diagnostic group is infants presenting with meconium ileus (MI) in the immediate postnatal period, accounting for up to 20% of new diagnoses. The remaining proportion are largely identified following clinical presentation with chronic respiratory or gastrointestinal symptoms, family history or through prenatal screening.

### 3.3. Clinical Characteristics

Given the multisystem progressive nature of the disease, the clinical characteristics of CF are extensive and range in severity, influenced by factors such as genotype and age. The most common clinical characteristics in the neonatal period are intestinal obstruction, EPI, faltering growth and recurrent respiratory infections [49,50]. As a child develops, respiratory morbidity can develop such as bronchiectasis and chronic pulmonary colonization by pathogens such as *Pseudomonas aeruginosa (PsA)* [51]; a pathogen strongly linked to accelerated lung disease and increased mortality in cwCF [49,52,53]. As pwCF age, other comorbidities related to CFTR dysfunction may arise alongside progressive lung disease, including CF-related diabetes and CF-related hepatobiliary disease, as well as an increased risk of noncommunicable diseases such as chronic kidney disease, CVD, overnutrition and gastrointestinal malignancies [2,4,5,21,54,55]. Phenotypic presentations of the disease are a direct result of pathophysiological processes related to the defective CFTR protein, combined with secondary complications due to the systemic effects of the underlying CFTR pathology, chronic inflammation, pharmacotoxicity and an ageing population [46,56]. The evolving nature of CF phenotypes is also due to the widespread uptake and expanding eligibility criteria of disease modifying drugs, known as CFTR modulators (CFTRm). The major cause of death for pwCF remains respiratory failure, although death from noncommunicable diseases is rising [3], highlighting the importance of preventative lifestyle interventions from an early age.

### 3.4. Nutrition and Lung Disease

Lung health is the major determinant of survival in pwCF [3,46,57]. Despite improvements in early life screening, therapeutic treatments and multidisciplinary care, nutrition continues to be an independent predictor of lung health and disease progression in children with CF [14,58,59,60]. Growth targets in the first 1000 days of life are centered around the achievement of a weight-for-length (WFL) at or above the 50th percentile; an outcome strongly predictive of optimal lung health [49,61,62,63,64,65]. These growth targets continue for children >2 years of age and persist into adulthood, where adults with CF continue to have a higher recommended BMI range than people without CF [61]. Although there is limited data on the upper limit of this range in terms of nutritional status and lung health in cwCF, there is some evidence to suggest that this benefit may exist only below the 85th percentile, above which point lung function begins to decline [60].

### 3.5. Evolving Treatments

CF care has evolved over the last decade from a largely downstream approach, where treatments focus on the consequences of CFTR dysfunction, to an increasingly upstream approach, where treatments such as CFTRm therapy target the production, intracellular processing or functionality of the defective CFTR protein. In recent years, the proportion of pwCF prescribed modulator therapies in the US, UK and Australia has surpassed 80% due to expanding variant and age eligibility [3,6,7]. The remaining proportion of pwCF who are ineligible are largely under the age of two years and this is due to ongoing clinical trials to establish safety and clinical efficacy to inform regulatory restrictions in this age group. This is an important point when examining early life nutrition and feeding in the first 1000 days of life, as evidence to date largely pertains to CFTRm naïve infants and children. CFTRm therapies have undeniably altered the trajectory of disease, contributing significantly to improved lung health, quality of life and a ten-fold increase in survival for pwCF [56,66,67]. However, there is also increasing evidence that they may be contributing directly or indirectly to the rising rates of overnutrition seen in modern day CF [68,69,70,71].

The evolving nature of therapeutic treatments, primary and secondary disease manifestations and a heterogenous population of CFTRm naïve and exposed children and adults, makes for a complicated picture when considering early life nutrition and feeding practices in the context of lifelong health and disease. The remaining sections of this narrative review will outline the key evidence relating to early life nutrition and feeding in the context of a rapidly evolving era of CF healthcare; generating hypotheses as to how the key nutritional components of the ‘first 1000 days’ framework may be applied to CF.

## 4. The Influence of Cystic Fibrosis on Growth and Nutrition During the First 1000 Days

For a cwCF, the first 1000 days of life can be marred by a myriad of complications, exposing them to further vulnerability within this critical developmental window [72,73,74,75]. Typical disease manifestations that present early in life for a cwCF and their influence on optimal growth and nutrition during the first 1000 days are outlined below.

### 4.1. Meconium Ileus

Meconium ileus (MI) is one of the earliest manifestations of CF, affecting up to 20% of infants with CF. Beginning in utero, MI is characterized by a build-up of viscid secretions within the terminal ileum, ultimately leading to small bowel obstruction. Simple MI, accounting for approximately 45–60% of cases, will present with a distended abdomen, bilious vomiting and dilated intestinal loops on imaging [76,77,78]. Complex MI reflects progression to volvulus, intestinal atresia, necrosis, peritonitis and/or perforation requiring surgical interventions including bowel resection and ileostomy placement [79]. Complications following surgery for MI are common, including sepsis and obstruction related to adhesions, and these can result in the need for subsequent surgeries, often leading to protracted hospitalizations [76,77,78,80]. Almost a quarter of infants born with MI are premature, further compounding neonatal complications [77,78].

Numerous studies within the last decade have demonstrated adverse medium to long-term health outcomes in infants diagnosed with MI compared to those diagnosed via newborn screening or clinical presentation, including reduced growth and lung function, higher and earlier rates of *PsA* acquisition and significantly higher mortality [77,81,82]. A retrospective multicenter Italian study by Padoan and colleagues [77] published in 2019 explored [77,78] the risk factors associated with poor 12-month clinical outcomes in 85 CF-MI infants. The authors found that 37% of the infants had poor outcomes at 12 months which included faltering growth and *PsA* infection [77]. Intensive care admission and cholestasis increased the likelihood of adverse events at 12 months of life [77]. A large retrospective registry study published by a Brazilian group in 2025 explored longitudinal clinical outcomes in 369 infants diagnosed with CF-MI and compared these to infants with non-MI CF [81]. The authors found that the CF-MI group had significantly lower weight-for-age and height-for-age z scores than the non-MI CF group and were more likely to be colonized with *PsA* in the first five years of life [81]. It is important to note that regional disparities in clinical care and access to timely diagnosis and key therapies was an acknowledged limitation of the study, potentially contributing to poorer outcomes in the CF-MI group [81]. Further, it is difficult to generalize findings from a LMIC, such as Brazil, where the quality of CF care is likely to be inferior to that of high-income countries, such as Australia, the UK and US. Although Tan and colleagues [82] in Australia also explored the outcomes of 161 matched infants with both CF-MI and non-MI CF and also found that the MI cohort had significantly lower BMI and lung function well into late childhood and early adulthood, but not *PsA* infection. Interestingly, earlier studies published between 2006 and 2010, which explored adverse outcomes in infants with CF-MI compared to infants with non-MI CF, did not show consistently poorer outcomes [83,84,85]. These findings may have been due to the non-MI groups being largely diagnosed via clinical presentation in the era before newborn screening was widely introduced; a time period associated with poorer outcomes [86], and should also be considered in the context of being conducted in high-income countries. The impact of MI on an infant’s cognitive, emotional or social development is unclear, with no known studies measuring neurodevelopmental or behavioral outcomes in this cohort.

With regards to feeding, there is very little evidence pertaining to breastfeeding rates in the CF-MI cohort. A small historical study published in 1991 by Holliday and colleagues [87] showed that infants with MI (n = 16) were less likely to breastfeed for at least three months, compared to their non-MI counterparts; 43.8% vs. 63.1%, respectively. Of the seven infants with MI who received breastmilk for at least three months, weight-for-age z scores at 12 and 24 months and length-for-age z scores at 24 months were significantly higher than the nine infants with MI who were predominately formula fed [87]. In the study by Padoan and colleagues [77], 54% of the CF-MI cohort (total n = 85) was breastfed for a median duration of 74 days, and there appeared to be a protective factor of breastfeeding against *PsA* infection and faltering growth at 12 months of life, although this result have been influenced by clinical complexity of the infant such as length of hospitalization and length of exclusive parenteral nutrition. For infants with complex MI requiring surgery, stoma formation, lengthy periods of exclusive parenteral nutrition and protracted hospitalization, breastfeeding may not be clinically appropriate, nor feasible from a maternal psychological or practical perspective, and as such, breastfeeding is unlikely to be the priority for these infants. Neonatal cholestasis, a further complication in these clinical scenarios, may also precipitate the need for specialized infant formula such as those with hydrolyzed proteins or high medium chain triglyceride (MCT) content [79].

### 4.2. Exocrine Pancreatic Insufficiency

The other major early extra-pulmonary manifestation of CF impacting early life nutrition is EPI, affecting more than 85% of infants and cwCF [46,88,89,90,91]. Extensive pancreatic damage occurs in utero resulting in destruction of the pancreatic acinar tissue [91] and symptoms of EPI, including growth faltering and feeding difficulties, can occur from as early as one month of age [49,90]. The cornerstone of treatment is pancreatic enzyme replacement therapy (PERT), typically porcine-derived enzyme preparations containing lipase, protease and amylase. PERT is provided to infants and young cwCF as enteric coated microsphere beads, typically fed to the child coated in a small volume of acidic puree. PERT is broadly speaking an effective treatment, although achieving optimal growth and symptom relief can be particularly challenging in the first year of life when growth requirements are at their highest and feeding patterns are changing frequently [90].

Despite improvements in the nutritional care of infants with EPI, pancreatic insufficiency remains strongly associated with adverse growth outcomes in infants with CF [14,15,92]. Recent observational studies in the US, UK and Europe show that infants with EPI are more likely to have lower birth weights [93,94] and reduced weight and linear growth in the first 1000 days of life [15,92,93], compared to infants with pancreatic sufficiency. The Baby Observational and Nutrition Study (BONUS) conducted in the US monitored the growth of 231 newly diagnosed infants with CF for the first 12 months of life and explored a host of clinical, gastrointestinal and pulmonary factors associated with growth outcomes [15,94,95,96]. Their findings show an increased risk ratio for low weight and low length in infants with EPI compared to infants with pancreatic sufficiency; 1.18 (95% CI 0.3; 4.64) and 2.25 (95% CI 0.59; 8.59), respectively [15]. EPI combined with MI increased this risk ratio for low weight and low length to 2.28 (95% CI 0.52; 10.07) and 3.42 (95% CI 0.83; 14.03), respectively [15]. Delayed linear growth has also been highlighted by Munck and colleagues [93], who demonstrated in their cohort of 99 newly diagnosed infants with CF that infants with EPI did not reach WHO median values for length-for-age until 24 months, compared to infants with pancreatic sufficiency who regained their small loss in length-for-age z scores by 6 months of age. Suboptimal linear growth is increasingly highlighted in the literature as a concern [95,97,98] and will be further discussed in subsequent sections of this review.

With regards to feeding, it might be reasonable to assume that infants with EPI are less likely to breastfeed due to early and pervasive gastrointestinal symptoms, particularly prior to establishing a therapeutic dose of PERT. However, there is conflicting evidence on this. In the BONUS and Munck cohort studies, as well as a recent cohort study of 172 newly diagnosed infants in the US known as the Feeding Infants Right from the Start (FIRST) study [14], infants with EPI were more likely to receive exclusive, predominate or partial breastmilk in the first six months of life than infants with pancreatic sufficiency [14,15,83]; contrasting to earlier studies showing the reverse [99,100]. This unexpected finding should be further explored in prospective newborn cohort studies and thought given to the design of qualitative studies which seek to understand parental experiences and attitudes to infant feeding in the first 12 months of life.

Whilst the consequences of EPI appear to act as a barrier to achieving optimal weight and linear growth within the first 1000 days of life for up to a quarter of infants and cwCF [92,93], the vast majority of infants successfully achieve catch up growth within this time period. Reassuringly, the impact of EPI on the ability to provide ‘any’ breastmilk may be less of a concern than historical data suggested, although rates remain well below those of typically developing children [13]. The evidence relating to the impact of EPI on other areas of development, outside of growth and nutrition, is limited.

With regards to the impact of CFTRm therapies on EPI, there is increasing evidence from clinical trials and short-term observational studies that pancreatic function may be restored, and EPI reversible in a subset of cwCF, although long term restoration or reversibility of EPI is unknown [101,102,103,104,105,106,107,108,109,110,111]. Greatest improvements are typically seen in infants with Rosenfeld et al. [109] showing EPI reversibility in 7 of 9 infants 12–23 months of age treated with ivacaftor for a period of 24 weeks with a mean (SD) improvement in faecal elastase of 164.7 (151.9) μg/g. Conversely, in the older age group, 2–5 years, Goralski et al. [103] showed only a 4% reversibility in their cohort of 75 children treated with elexacaftor/tezacaftor/ivacaftor for a period of 24 weeks with a mean (SD) improvement in faecal elastase of 39.5 (89.2) μg/g. Notably, these trial-based studies are short-term and longitudinal studies exploring sustained exocrine pancreatic restoration are required. Further, in vivo animal and human studies of fetuses with CF treated prenatally with CFTRm therapy have shown the preservation of pancreatic function at birth; however, again, we are unsure of what happens clinically beyond the neonatal period [112,113,114,115]. To date, it remains unclear as to the extent that factors such as genotype, CFTRm drug and age at commencement predict pancreatic restoration following modulator therapy. Further phase 3 clinical trial evidence and robust longitudinal observational data are required to provide definitive evidence of efficacy for CFTRm therapy on pancreatic restoration, either in utero or early in life. Until such evidence is available, EPI will continue to impact nutrition in most cwCF within the first 1000 days of life.

### 4.3. Other Gastrointestinal Manifestations

Gastrointestinal manifestations of CF are also complex and multifactorial in nature, relating in part to the altered pathophysiology of the defective CFTR gene and the resulting influence on intestinal acidity and luminal secretions, dysmotility and intestinal inflammation [116,117,118,119]. Widespread and pervasive gastrointestinal disorders appear prior to the onset of respiratory disease in infants and cwCF and those most likely to affect the younger population include gastroesophageal reflux (GOR), constipation and intestinal obstruction. Hepatobiliary complications, although less common, may also arise during infancy and childhood, further compromising growth and nutritional status.

Dysbiosis is also increasingly recognized as a significant disease burden for pwCF, beginning early in life with infants showing reduced levels of beneficial bacteria, increased opportunistic pathogens, decreased richness and diversity of intestinal microbiota and delayed maturation of the microbiome [95,120,121,122,123,124,125,126]. The dysbiotic gut of infants and cwCF is associated with poor outcomes such as local and systematic inflammation, compromised immunity, *PsA* infection and reduced linear growth [95,120,121,122,123,124,125,126]. Modulation of the gastrointestinal microbiome is influenced in part by diet; a heavily researched topic in the general population [127,128]. Evidence pertaining to dietary modification of the dysbiotic CF gut will be discussed in Section 5.4 of this review.

Reflux is seen in 46% of infants and cwCF, according to a 2020 systematic review of GOR in cwCF [129], with research indicating it may be associated with reduced lung function, earlier acquisition of *PsA* and poor growth, particularly if left untreated [130]. It is well recognized that many cwCF do not present with typical symptoms of GOR [130,131,132], such as heartburn and regurgitation, with children more commonly presenting with symptoms such as abdominal pain and chronic cough. Infants are even more difficult to diagnose, with a lack of overt symptoms [130,133].

Constipation is a chronically underdiagnosed and debilitating condition in cwCF with an estimated incidence of 47% in children [134]. In the BONUS study, infants were found to experience constipation, based on reported stool consistency, at a frequency of 10.8–12.9% in the first year of life [135]; however, rarely required hospitalization for management [96]. Distal intestinal obstruction syndrome (DIOS), whilst sharing some phenotypical characteristics with constipation [136,137], is a distinct condition unique to CF that is characterized by a build-up of inspissated mucus at the terminal ileum. DIOS can present as a partial or complete bowel obstruction and carries a higher risk of morbidity and protracted hospitalization than constipation [119,136,137]. DIOS is thought to occur in 7–8% of cwCF and is more likely to occur in children with certain genotypes, EPI, previous laparotomy and neonatal MI [119,136,137,138,139]. The incidence of DIOS across childhood appears to be evenly distributed [138] although there is limited evidence on the incidence or prevalence of DIOS in the first two years of life specifically. Growth outcomes in children with chronic constipation and recurrent DIOS are not well understood although both conditions can present with several risk factors for poor nutritional intake including abdominal pain, high doses of osmotic laxatives and hospitalization. The BONUS study recently demonstrated that gastrointestinal-related hospitalization within the first year of life had the greatest negative effect on weight and length, as compared to respiratory-related hospital admissions [96]. In their findings, 60% of gastrointestinal admissions were for malnutrition with the longest admissions related to reflux [96].

The long-term impact of early life gastrointestinal manifestations in CF such as dysbiosis, GOR, chronic constipation and DIOS are poorly understood. Evidence from non-CF populations tell us that the critical window of opportunity for establishing life-long microbial diversity is the first several years of life, and that disruptions to this process can have lasting health effects, including an increased risk of obesity, inflammatory and autoimmune conditions and cancers [140,141]. However, the recent literature suggests that the microbiome can continue to be diversified well into adulthood [142,143], providing opportunities for targeted interventions. Again, evidence from non-CF populations suggests the risk of gastrointestinal cancers is elevated in people with chronic reflux and intestinal inflammation [118,144,145]; conditions which infants and cwCF experience at higher rates than the healthy population. Opportunities to intervene early, or to modulate these risks with CF-specific evidence-based dietary therapies are, therefore, crucial. Unsurprisingly, gastrointestinal health is a top research priority for pwCF and treating clinicians due to the overwhelming impact on health outcomes and quality of life [146].

The impact of CFTRm therapies on gastrointestinal health and the prevalence of abdominal symptoms in pwCF is emerging. However, given the eligibility restrictions in children less than two years, there is limited evidence for the impact of CFTRm therapy on gastrointestinal health in this age group. In school-aged children and adolescents, ETI has been shown to improve appetite, constipation and abdominal pain [147], as well as increasing microbial diversity, the presence of beneficial intestinal species and reducing intestinal inflammation [148,149,150]; although longitudinal studies are lacking. With regards to the incidence of DIOS following CFTRm therapy, to the best of our knowledge, there is no data in the paediatric population, although there is some evidence that in adults, the incidence may be reduced [151]. Longitudinal observation of DIOS presentations in cwCF exposed to early life CFTRm therapy is important as there is the potential that early CFTRm treatment may confer a greater benefit on intestinal manifestations, compared to later life CFTRm exposure.

### 4.4. Compromised Nutrition

Compromised nutritional status is the result of these hallmark gastrointestinal and pancreatic features of CF. Whilst significant inroads have been made to reduce the prevalence of undernutrition in CF over the last several decades, reportedly falling by as much as 40% [152], undernutrition remains strongly associated with a higher risk of morbidity and mortality and a reduced quality of life for pwCF [9,10,46,61,75,153,154,155,156]. Infants and cwCF, due to their unique growth requirements and the host of factors predisposing them to suboptimal nutrition, are particularly vulnerable to the systemic effects of malnutrition [49,61,93,156].

Energy requirements for infants with CF may be as much as 120–150% above estimated requirements for typically developing infants, largely due to the metabolic demands of chronic inflammation and early life lung disease counterbalanced with an energy deficit from EPI-related malabsorption [49]. Increased energy requirements in infancy are typically achieved through breast milk fortification or infant formula supplementation and/or concentration [49,61]. In the BONUS study, 40% of infants who were exclusively formula fed received additional energy through concentrated formula preparation at 3 months of age, increasing to 52% at 6 months of age [15]. Similarly, 53% of infants in the FIRST study were receiving additional energy supplementation, through either breastmilk or infant formula fortification, by 3 months of age, increasing to 64% by 6 months of age [14]. The most recent European CF nutrition guidelines do not specifically recommend energy targets for infants with CF, instead taking a less prescriptive stance by recommending increased energy provision “only as necessary for the first year of life” [61]. There is no evidence to date on how CFTRm might alter energy requirements in infancy, but it would be reasonable to assume that the results would be highly variable, depending on the degree of lung impairment and exocrine pancreatic restoration.

Despite their nutritional vulnerability, recent registry data from countries such as Australia and the US, show that on the whole, cwCF are achieving optimal growth in first two years of life [3,7]. The 2023 Australian CF Data Registry Report indicates that the median WFL percentile for infants and children less than two years of age is the 54th percentile, whilst for children older than two years of age, the median BMI percentile is the 60th [7]. The 2024 US CF Data Registry Report shows similar median percentiles [3]. Whilst this is an indication of nutritional success for the majority of infants and cwCF, 11–15% of infants and cwCF remain undernourished at two years of age and as many as 24% stunted using definitions of weight-for-age and length-for-age less than the 10th percentile [3,6,7,15,92,93,157]. Head circumference, although not as widely described in the growth literature, was reported as normal at 3, 6 and 12 months of age in the BONUS study. Registry data, along with cohort studies such as BONUS and FIRST, highlight that growth in cwCF is still a concern within the first 1000 days of life, albeit for a smaller proportion of children, and that factors such as birth weight, EPI, MI and *PsA* infection are associated with suboptimal nutrition during this period [14,15,92,93].

Focusing specifically on reduced linear growth in infants and cwCF, there is increasing CF-specific evidence that stature is not wholly related to nutrition and genetic potential and that final adult height may be determined by factors such as linear growth within the first 1000 days of life, male sex, intestinal dysbiosis, pancreatic phenotype and genotypes associated with more severe disease [15,95,158,159,160]. Stunting in cwCF is strongly associated with reduced longitudinal pulmonary health and increased mortality [10,15,98,161,162,163,164]. Optimization of linear growth is therefore a priority in the first 1000 days of life and may serve as an important independent outcome when measuring success of early life CFTRm therapy. In children without CF, stunting in the first two years of life is associated with both short and long-term consequences including neurocognitive delays, increased susceptibility to infections, the accumulation of visceral fat and the development of noncommunicable diseases such as type 2 diabetes and CVD [165]. Similar to the non-CF population, studies in cwCF show that stunting often occurs despite normal WFL or BMI status [15,166]. In the BONUS study, 53% of infants who were stunted had normal weight-for-age z scores. Many studies do not report length or height-for-age as an independent marker of nutritional status, thereby failing to identify a cohort of children who may have suboptimal linear growth [166]. The continued emphasis on WFL and BMI equal to or greater than the 50th percentile may inadvertently contribute to clinicians overlooking inadequate linear growth and the influence that this might have on health outcomes such as lung health, despite clear recommendations in nutrition guidelines for the regular assessment and monitoring of linear growth [61]. Ultimately, comprehensive nutritional assessment in cwCF across the first 1000 days of life must include a range of anthropometric parameters, interpreted together and routinely assessed over time [61]. Furthermore, future nutrition-focused research in CFTRm naïve and exposed populations should consider assessment of linear growth as a primary endpoint, alongside WFL and BMI.

Optimization of lung health in the first 1000 days of life is inextricably linked to nutritional status with compromised nutrition adversely affecting early life pulmonary outcomes. Two recent large CF data registry studies from the US (n = 6809) and UK/Canada (n = 2765) confirmed the strong relationship between early life nutrition and lung health [58,59]. To summarize their combined findings, the children with the highest lung function at 6 years of age demonstrated a WFL or BMI consistently above the 50th percentile. Whereas children with the lowest lung function demonstrated a WFL or BMI below the 25th percentile or, a consistent deceleration in growth. The findings of these two recent and extensive registry studies are consistent with the previous literature, unquestionably showing the strong association between nutrition and lung health in cwCF and the influence that growth trajectories in the first 1000 days of life have on pulmonary outcomes in later childhood. Historical and current nutritional recommendations are built on this evidence, with the goal to achieve a WFL and BMI at or above the 50th percentile, with values less than the 25th percentile considered ‘nutritional risk’ [49,61].

Growth outcomes in infants and cwCF less than two years who are receiving CFTRm therapy demonstrate improvements in all growth parameters in phase 3 clinical trials, with the magnitude of absolute change in parameters depending on age. Infants 1–4 months, 4–12 months and 12–24 months treated with ivacaftor for 24 weeks showed improvements from baseline in weight, length and WFL z scores, most notably in the 1–4-month and 4–12 month age groups [102,105,109]. Of note, absolute change in length-for-age z scores after 24 weeks of therapy were 1.12, 0.37 and 0.28 for infants 1–4 months, 4–12 months and 12–24 months, respectively; representing clinically significant changes in linear growth for these children. The potential for early life modulator therapy to support linear growth within the first 1000 days of life is particularly exciting given many studies in older children receiving CFTRm therapy show no significant changes in height z scores compared to pre-modulator baseline values [103,107,167,168]. The first 1000 days could represent an important window for optimization of linear growth, particularly in the context of CFTRm therapy.

As a final note on compromised nutrition, it is important to also highlight the double burden of malnutrition that exists in CF; that is, the increasing prevalence of overweight and obesity in older children and adults with CF. The prevalence of overweight and obesity in cwCF has surpassed 15% in high-income countries [7,152], and the US has seen a 300% and 400% increase in the proportion of pwCF with overweight and obesity, respectively, during the period 2000 to 2019 [152]. This US CF data registry study reported key patient demographic and clinical variables associated with an increased risk of overweight and obesity, of which included having at least one copy of a mild CFTR mutation, older age, treatment with CFTRm therapy and living in an area with low median household income [152]. Environmental factors likely to be associated with the increasing prevalence of overweight and obesity in the CF population are a lifelong exposure to abundant energy and dietary fat intake; however, aside from geographical location, these were not assessed in this US registry study. Proposed mechanisms of CFTRm-associated weight changes include reduced resting energy expenditure, changes to body composition, most notably increased fat mass, and improvements in exocrine pancreatic function and intestinal inflammation [68,69,70,152,169]. Early life nutritional programming may also be a consideration: historically high rates of infant and early childhood malnutrition in CF, in combination with an emphasis on high energy diets may have contributed to the evolving picture of overweight, obesity and cardiometabolic conditions that we now see in the CF population, although this is purely speculative. Despite the unique physiological and metabolic milieu of CF, DOHaD may be a relevant framework to consider in modern day CF.

## 5. The Influence of Cystic Fibrosis on Early Life Feeding During the First 1000 Days

Early life feeding practices, including the establishment of breastfeeding, are crucial elements in a child’s growth and development and in the attainment of lifelong health and wellbeing, and in cwCF, are thought to be vastly different to that of typically developing children [13,14,15,16,170]. The foundational pillars of the ‘first 1000 days’ for optimal early life nutrition include quality prenatal nutrition, exclusive breastfeeding and nutrient-rich complementary feeding and are explored in the context of cwCF below [171].

### 5.1. Prenatal Nutrition

The lifelong programming of health and disease, particularly non-communicable diseases, begins prenatally; a concept well-described in the literature [17,44]. The importance of a mother’s preconception nutritional status and her diet during pregnancy is well-acknowledged with maternal undernutrition, overnutrition and diet quality influencing maternal and perinatal outcomes, early development and lifelong health and disease [44,172]. In the non-CF population, mothers who have overweight or obesity are more likely to experience complications during pregnancy and delivery and their babies are more likely to be low birth weight or suffer congenital abnormalities [44]. Mothers without CF who are undernourished are more likely to have babies who are premature, small for gestational age and who present with neonatal complications such as infection [17,44,173]. The importance of a diet rich in vitamins, minerals and trace elements during pregnancy and specially formulated prenatal micronutrient supplements is universally recognized, with key micronutrients such as folate, iodine, iron, choline, calcium, vitamin D and polyunsaturated fatty acids (PUFAs) essential for optimal fetal growth and development [174]. In the non-CF population, prenatal nutrition counselling has been shown to improve nutritional status and healthy eating of expectant mothers and may have an impact on maternal and neonatal health outcomes [175,176]. Likewise, prenatal breastfeeding education may have a positive impact on breastfeeding initiation, duration and exclusivity in the non-CF population [176,177]. Opportunities for preconception and pregnancy nutritional counselling in the broader population are limited by factors such as timely access to quality prenatal care, including access to appropriately trained nutrition experts.

When looking at the role of prenatal nutrition in CF, it is important to note that the number of infants diagnosed with CF prenatally is very low, approximately three percent [7,157], thereby limiting the scope to modify this key nutritional pillar within the first 1000 days of life. A prenatal diagnosis of CF is typically made following pre-pregnancy genetic carrier screening, prenatal testing or fetal ultrasound findings consistent with CF [178]. Within the context of CF and the first 1000 days of life, there are two key groups to consider when describing preconception nutritional status or opportunities for prenatal nutritional intervention: (1) women without CF whose infants are diagnosed with CF prenatally and (2) women with CF whose infants are diagnosed with CF prenatally; the latter being more nutritionally complex. For women without CF where the infant’s diagnosis is made early in the pregnancy, referral to a nutrition expert can be expedited, although in practice, this rarely occurs. Ideally, multidisciplinary team input from a specialized CF center would occur prenatally for these mothers, as soon as the diagnosis is confirmed, providing among other things, an opportunity for specialized pre and postnatal nutrition.

In practice, opportunities for personalized prenatal nutrition counselling and intervention are enhanced in women with CF as the mother should already be engaged with an appropriately skilled dietitian as part of her multidisciplinary CF care. CwCF born to mothers with CF are a particularly vulnerable group given the double burden of maternal and child malnutrition risk. Early intervention might be most beneficial in this cohort, where specialized dietary advice can be tailored to the mother, from preconception to pregnancy and breastfeeding; paying particular attention to the micronutrient needs of both the mother with CF and the unborn child with CF. Pre-existing maternal nutritional deficiencies, or micronutrient deficiencies carried throughout the pregnancy will impact the micronutrient status of the infant with CF, including the provision of key nutrients in breastmilk such as iron and PUFAs. Prenatal breastfeeding education for women with and without CF, and planning for early specialized lactation support may assist in breastfeeding exclusivity and duration for prenatally diagnosed infants with CF, where otherwise this opportunity would have been delayed. Finally, although the risk of malnutrition for pregnant women with CF is high [49,61], recent data shows us that the pre-pregnancy BMI status of women with CF who fall pregnant is typically healthy, with a median BMI in the range of 21–22 [179,180]. Optimized preconception nutritional status in CF is likely due in part to the substantial emphasis in recent decades within the CF healthcare system on pre-pregnancy nutritional optimization for improved maternal and neonatal CF outcomes [49,61].

### 5.2. Breastfeeding

Breastfeeding rates in the CF population are markedly reduced compared to the general population, with a 2021 systematic review and more recent observational cohort studies indicating that exclusive breastfeeding rates are at least half that of typically developing children at 3 and 6 months of life [13,14,15]. Early supplemental infant formula use is high, and by 3 months of age as many as 56–71% of infants are exclusively fed infant formula [15,93,94]. Knowledge of determinants of successful breastfeeding in CF is limited. The FIRST study reports that, similarly to the general population, higher levels of education and income positively influence breastfeeding duration in the CF population [14]. Conversely, maternal-reported stress, burden of care and inadequate specialized lactation support are thought to be contributing factors to early breastfeeding cessation in CF [181,182]. A small mixed methods study by Miller et al. explored the benefits of early lactation support integrated within the first several newly diagnosed CF appointments in a group of 17 mother-infant dyads and compared breastfeeding exclusivity and duration with a historical control group who did not have access to integrated and early lactation support [181]. Both breastfeeding exclusivity and duration were increased in the lactation support group, highlighting an important opportunity for early intervention. Whilst not described in the CF literature specifically, it is likely that the introduction of supplemental infant formula, beyond the neonatal phase, will have an impact on both maternal milk supply and breastfeeding duration given what we know from evidence in non-CF populations [183,184]. A 2021 systematic review on breastfeeding challenges in medically complex children revealed that there were a range of unique challenges with regards to the sustainability of breastfeeding in these infants: practicalities of the infant’s hospitalization, psychological parental distress, the impact of acute critical illness and surgeries, the impact of chronic illness and symptoms, the availability of specialized lactation support in the hospital setting and the breastfeeding attitudes of healthcare professionals caring for their infant [185]. All of these challenges described are applicable to the CF setting.

CF nutrition guidelines have long recommended exclusive breastfeeding for the first six months of life, aligning with WHO recommendations for all infants, with breastmilk to continue alongside the introduction of complementary foods for up to two years [49,61,63,65,186]. Caveats do exist in the guidelines, however, for the introduction of breastmilk fortification or supplementary infant formula for the prevention and treatment of suboptimal growth [49,61,63,64,65,186,187].

Studies as early as 1990 have demonstrated the potential benefits of breastfeeding on pulmonary health for cwCF [13,14,15,61,77,87,93,99,100,123,187,188], although their findings often limited by small sample size, retrospective data collection and failure to account for potential confounding variables. Recently, the FIRST study reported that proportionally, significantly fewer infants fed predominately fortified breastmilk had moderate and severe early lung disease, compared to infants fed predominately fortified infant formula or a combination of both (30% vs. 62% and 53%, respectively); after adjusting for factors such as pancreatic phenotype, CFTR genotype and CFTRm therapy [14]. Severity of lung disease in the FIRST study was assessed using a previously published scoring system [189], and included a combined assessment of respiratory symptoms, pulmonary exacerbations, hospitalizations and *PsA* infections [14]. However, as isolated outcomes, no significant differences were found in the number of pulmonary exacerbations or positive *PsA* infections in either the unfortified or fortified breastfed groups compared to formula fed groups [14]. Taken as a whole, it is likely that those infants with minimal lung disease by three years of age benefited most from the combined action of fortified feeds to ensure optimal growth, alongside the unique bioactive properties of breastmilk.

The mechanisms for proposed benefits of breastmilk on pulmonary and extrapulmonary health are thought to include the immune modulating properties of breastmilk on infection and inflammation, the influence of breastmilk on microbial diversity in the respiratory and gastrointestinal tract and the provision of essential fatty acids (EFAs) which have strong anti-inflammatory and immune-mediating properties and an important role in cell membrane fluidity and permeability [65,123,190,191]. With regards to the influence of breastmilk on microbial colonization of the respiratory and gastrointestinal tracts and clinical outcomes, a small prospective observational study of 13 newly diagnosed infants reported increased respiratory and gastrointestinal microbial diversity and delayed time to first respiratory exacerbation when infants were exposed to any breastmilk, as opposed to exclusive infant formula [123]. Whilst the application of the author’s findings is limited by the study’s small sample size, it nicely highlights future opportunities for larger studies to further explore breastmilk exposure, exclusivity and duration on clinical outcomes for the infant with CF, strengthening the evidence on pulmonary health and eliciting unexplored benefits on extrapulmonary health.

Growth outcomes in infants fed exclusive or predominate breastmilk compared to those fed exclusive infant formula are more difficult to interpret. The FIRST study reports significantly lower longitudinal BMI z scores from birth to 36 months for infants fed unfortified breastmilk, in comparison to those infants fed unfortified infant formula, or a combination of both [14]. However, it is important to note that by 12, 24 and 36 months, despite the breastfeeding group having lower BMI z scores than the infant formula group, values were still above the recommended 50th percentile. In addition, when fortified, the breastmilk group achieved similar BMI z scores at 6, 12, 24 and 36 months, as compared to the fortified infant formula group [14]; both groups achieving a BMI greater to or equal than the 50th percentile at all time points combined with superior pulmonary outcomes, as discussed above [14]. Further to this, three prospective longitudinal cohorts published in the last decade, including the BONUS study, have reported that mode of feeding has no predictive value on growth outcomes at 12 and 24 months [15,92,93]. BONUS infants fed exclusive breastmilk achieved a higher weight-for-age z score at 6 months than those fed exclusive infant formula; however, by 12 months, both groups were similar, approaching the 50th percentile [15]. What does appear to be consistent, at least in the BONUS and FIRST cohorts, is rate of weight gain in the first 12 months. The rate of weight gain for infants with CF fed exclusive infant formula appears to be higher than that of infants fed exclusive breastmilk [14,15]. A potential consideration when examining the association between formula feeding and weight trajectory is that infants requiring exclusive infant formula from birth may be clinically unwell, resulting in an initial decline in weight followed by significant catch-up growth aided by concentrated infant formula.

The universal benefits of breastmilk on an infant’s growth, development and later life health and disease are critical to consider in the context of CF, a disease of chronic infection and inflammation. The bioactive components of colostrum and breastmilk play a crucial immunological and anti-inflammatory role in the first few months of life [192], roles which may be critical for an infant with CF. Antibodies from breastmilk, predominately IgA, function primarily in the mucosal lining of the gastrointestinal and respiratory tracts, acting as an infant’s first line defense against infectious diarrhea, necrotizing enterocolitis and acute respiratory infections [192]. Lactoferrin, with its immune modulating and anti-infective properties is known to protect against common respiratory and gastrointestinal infections in non-CF children, as well as potentially influencing gut microbiome diversity [193]. An abundance of anti-inflammatory cytokines present in breastmilk, are important for the lifelong programming of the body’s immune system [192]. Human milk oligosaccharides (HMOs), resistant to enzymatic action in the upper gastrointestinal tract, pass through to the colon where they play a crucial role in the modulation of the gut microbiome [192,194,195]. In a disease characterized by dysbiosis and an abundance of pro-inflammatory-associated gut bacteria [196], the action of biological compounds such as HMOs and lactoferrin in breastmilk are likely to be important for early life modulation of the gut microbiome in CF [95,123,124]. Indeed, it is increasingly evident in the broader literature that breastfeeding is a key modifying factor of an infant’s developing microbiome structure, particularly with regards to the richness of health-promoting bacteria such as *Bifidobacteria* [197,198,199,200].

Children and adults with CF who carry genotypes associated with more severe disease are known to have low levels of circulating EFAs; specifically, linoleic acid, alpha linolenic acid and DHA [190,191,201,202,203,204,205]. EFA deficiencies in CF are thought to be directly related to CFTR dysfunction and the subsequent impact on cellular fatty acid metabolism and lipid homeostasis [201,202,206]. EFA are polyunsaturated fats which are unable to be synthesized by the body and must be obtained from dietary sources. PUFAs, present in large quantities in human milk, are required for cell membrane fluidity, integrity and function, particularly in the brain, retina and nervous system [207]. In addition, PUFAs serve as precursors for bioactive lipid mediators which regulate inflammatory and immune pathways; DHA being particularly important in the suppression of pro-inflammatory markers [206]. In cwCF, EFA deficiencies have been linked to poor growth and pulmonary health [208] and it is postulated that human milk PUFA content may help to supplement underlying EFA deficiencies in infants with CF [206].

### 5.3. Infant Formula

A large proportion of infants and cwCF receive infant formula, either supplementary to breastmilk, or as a breastmilk replacement. By the third month of life, when peak growth faltering is escalating, more than 50% of infants with CF are exclusively formula fed [14,15,93,94]. The decision to commence supplementary infant formula may come from the CF team in response to faltering growth or severe EPI, or may be a maternal-driven decision, as was recently described in Colborg et al.’s qualitative study on the barriers and facilitators to breastfeeding cwCF [182]. The type of infant formula will depend on the clinical characteristics of the infant with CF and the reason for commencing infant formula, as well as maternal preferences; however, in practice, most infants with CF will tolerate a standard cow’s milk based infant formula [49,61,65]. A smaller proportion may require extensively hydrolyzed, amino acid or high MCT formulas to assist with severe malabsorption or recovery from bowel surgery.

Colborg et al. interviewed mothers of cwCF about their recalled experiences breastfeeding their cwCF; key themes included mothers feeling that formula feeding was better for their infant due to the perceived benefit of “extra calories”, being able to measure intake for “peace of mind” and for relieving stress related to poor growth [182]. Conversely, some mothers also expressed immense disappointment, guilt and sadness when they were recommended to commence supplemental infant formula [182]. The authors concluded that clinicians should be cognizant of predetermined maternal feeding preferences, CF specific barriers to breastfeeding exclusivity and duration and to tailor nutritional interventions to maternal feeding preferences and goals, as well as to the infant’s clinical needs.

Evidence from large cohort studies and randomized trials in children without CF demonstrate that infants who are fed predominately infant formula, as opposed to breastmilk, reach a significantly higher weight-for-age and WFL at 12 months compared to their breastfed counterparts and are more likely to develop overweight and obesity by school age [209,210,211,212,213]. It is also evident in infants without CF that those with rapid weight gain, generally defined as a change in weight-for-age z score of >0.67 (>25 percentile points), are more likely to be overweight as children and adults [214,215,216]. These associations between early life nutritional exposure and growth patterns are examples of metabolic programming embedded in the DOHaD framework. As discussed in the CF literature, the BONUS study demonstrated that growth outcomes at 12 months of age in cwCF fed exclusive infant formula are largely similar to those fed exclusive breastmilk [15]; similar results in the FIRST study with the caveat of both groups being energy supplemented [14]. However, rate of weight gain in infants with CF fed exclusive infant formula appears to be more rapid in the first 12 months of life when compared to those fed exclusive breastmilk [14,15]. What is unknown and posed hypothetically, is if infants with CF who are fed exclusive or predominate infant formula, or who experience rapid weight gain in the first 1000 days of life, are more likely to be overweight or obese as adults. Given the known association between early life formula feeding and later life obesity risk in the non-CF population, alongside the high proportion of formula fed infants with CF and the increasing rates of obesity in children and adults with CF, this hypothetical association needs to be explored in the CF context. Whilst the physiological and metabolic underpinnings of CF complicate this question, longitudinal studies exploring associations between early life feeding practices and growth trajectories, and later life metabolic complications would be of great benefit to further refine early life nutritional recommendations.

Alterations to the macronutrient content of infant formula, such as reduced protein content which has been widespread in recent years, may alleviate some of this rapid weight gain risk in formula fed infants [209]; however, the physiological and behavioral mechanisms involved in breastfeeding compared to bottle feeding are likely to still play a significant role in feeding practices and intake between breastfed and formula fed infants [216]. Bottle feeding, regardless of whether it is expressed breastmilk or infant formula, is more likely to be associated with rapid weight gain in infancy than breastfeeding due to a number of proposed reasons such as impaired self-regulation and parental overfeeding [216]. It is quite plausible that in the CF population, parental overfeeding due to a hyperfocus on feeding schedule and volumes consumed, alongside either perceived or real growth concerns, puts infants with CF at a higher risk of overfeeding and rapid weight gain than infants who are breastfed.

### 5.4. Complementary Solids

The timely introduction of nutrient-dense complementary foods is a key component to the ‘first 1000 days’ concept. WHO recommends that infants should be introduced to nutrient dense, rather than energy dense foods, with the goal to learn to accept healthy foods and establish long-term optimal dietary patterns [217]. It is well established in non-CF populations that inappropriate complementary feeding, with excessive energy dense and nutrient poor foods, can result in later life overweight, obesity and chronic disease [214,218,219]. Furthermore, diet quality, the balance or variety of nutrient-dense eating patterns and foods, is recognized as an important modifiable risk factor for chronic disease and mortality [220]. Globally, only 28% of children under two years of age meet minimum dietary diversity standards and infants 6–11 months of age have the lowest dietary diversity compared to children in other age groups [221].

From a CF perspective, the most recent European nutrition guidelines recommend that complementary solids should be introduced to the infant with CF at the same time as other children; that is, greater than four months of age and not delayed beyond six months of age [61]. This has been consistent advice in CF since at least the early 2000s [62,63,65,222]. To date, however, very little guidance can be found on the composition of complementary foods for infants with CF, other than to ensure appropriate energy for normative growth. In practice, CF dietitians follow WHO and region-specific early feeding guidelines, adjusting macro- and micronutrient content during the complementary feeding period as clinically required.

Until recently, the ‘CF legacy diet’, high in energy, fat and salt, was recommended for all pwCF from the age of complementary solids, with recent guidelines advising energy and fat restriction only in the case of overweight or obesity [49]. It wasn’t until the publication of the 2020 US CF nutrition guidelines that high fat diets were discounted as a therapeutic diet beyond meeting energy targets [186]. Guidelines now recommend dietary fat intakes for pwCF in line with general population recommendations [61]. The quality and source of dietary fat intake however have been discussed in historical and recent guidelines, with an emphasis on the inclusion of unsaturated fat and EFAs [62,63,65,222]. As highlighted earlier in the review, EFA disturbances and deficiencies and their associated impacts on health outcomes for pwCF are an ongoing concern in the CF population; nutritional recommendations to increase dietary intake of EFAs aim to address this concern, although they do not explicitly recommend routine supplementation due to insufficient evidence [49,61]. In reality, the diets of pwCF continue to be dominated by high statured fat intake with excessive energy-dense and nutrient-poor food choices [49,203,223,224,225,226,227,228].

Diet quality is a concept that was lacking from nutrition guidelines until the 2017 Australasian CF nutrition guidelines [49]. More recently, the 2024 European CF nutrition guidelines alluded to the benefits of diet quality but did not feature it as a key concept [61]. The publication of recent systematic reviews, cross-sectional and interventional studies in children and adults with CF has placed diet quality firmly in focus, with the scientific and clinical community advocating for a high-quality diet from early childhood [226,227,229,230]. Furthermore, the CF legacy diet is now in the spotlight with regards to the contribution it may have had on the development of rising chronic and metabolic disease in pwCF [69,228,231]. The importance of diet diversity and quality in CF encompasses many aspects of emerging health concerns for pwCF, including the prevention of later life noncommunicable disease, modulation of inflammatory process and optimization of microbial diversity. Whilst individual micronutrients such as calcium, iron and zinc remain important to assess individually, a whole-food approach to the therapeutic CF diet is gaining traction, prioritizing the balance of essential nutrients and macro and micronutrient quality over macronutrient distribution [226,227,230,232]. Individualization of the diet is key, particularly for individuals who are malnourished, ineligible for CFTRm therapy or who have additional comorbidities such as CF-related hepatobiliary disease.

There are an increasing number of diet quality and diversity studies in both pediatric and adults CF populations, although limited evidence in infants with CF [122,226,227,228,229,230,233,234,235,236]. The GreeCF study looked at adherence to CF dietary guidelines and overall diet quality in a cohort of school aged cwCF in Greece and found that 75% of children had poor or moderate diet quality [236]. Key dietary quality themes included excess energy and saturated fat, low dietary fiber and low intakes of essential fatty acids and vitamin A [236]. Likewise, an Australian group looked at the dietary quality of cwCF 2–18 years and compared their intake to age and sex-matched healthy controls [234]. These authors found that after adjusting for energy intake, which was significantly higher in cwCF, micronutrient density in cwCF was significantly lower than healthy controls, with the exception of vitamin A, calcium, phosphorous and sodium [234]. An earlier cross-sectional study of dietary intake in cwCF 2–18 years by the same group of authors revealed that cwCF consumed significantly more energy-dense and nutrient-poor foods than their non-CF counterparts [235]. A recent prospective cross-sectional study, also from an Australian group, observed the dietary intake and quality of 41 cwCF 0–18 years compared to healthy counterparts, and in the CF group, explored associations with dietary intake and gastrointestinal and respiratory microbiota, intestinal inflammation and clinical outcomes [122]. Poor dietary quality was observed in the CF group, compared to healthy controls, and similar to previous studies, cwCF consumed more energy and total fat, less dietary fiber, less wholegrains and resistant starch and less dietary iron compared to healthy controls [122]. It is important to note that whilst infants and young children were eligible for inclusion, the median (IQR) age of participating children was 9.2 (5.7, 13.5) years; therefore, poorly representative of the 0–2 year age group. Overall, these studies reveal a consistent trend towards poor diet quality in cwCF, in addition to high energy and dietary fat intakes relative to children without CF. They also highlight the pronounced evidence gap that exists with regards to complementary food patterns and diet quality in the first 1000 days of life for cwCF.

The modifiable action of complementary foods on an infant’s gastrointestinal microbiota is limited in the CF literature, with most studies either describing microbiome diversity in infants and cwCF or looking at associations between the gastrointestinal microbiome and CF-related pulmonary and growth outcomes [95,121,123,124,125,237,238]. Conversely, there are an increasing number of observational studies in the general population which explore the influence of complementary solids, including type and diversity of solid foods introduced, on early life microbiota diversity and maturation and long-term health outcomes [198,200,239,240,241,242,243]. A single small observational study described the composition of fecal microbiota in six exclusively breastfed infants with CF and documented changes following the introduction of solids foods in three of these infants [239]. No definitive conclusions could be drawn from this study owing to small numbers and large heterogenicity of bacterial species between individual infants, but the authors suggest there would be benefit in further larger studies establishing the effects of individual foods and complementary feeding practices on health-promoting microbiota diversity and maturation in cwCF [239]. Such studies are particularly important in CF where dysbiosis is a significant gastrointestinal problem [121,122,123] and the optimization of nutrient dense complementary foods may prove an opportunity to modify microbiome diversity, maturation and functionality.

### 5.5. Parental Burden

Parents and caregivers of cwCF are known to experience high levels of mental health concerns, with up to 50% experiencing anxiety symptoms and up to 21% experiencing depressive symptoms [244,245]. Nutritional cares and the stress of feeding a cwCF contribute significantly to this burden [182,246,247,248,249]. A bidirectional relationship between child feeding behaviors and parental stress, coping strategies and parenting styles at feeding and mealtimes is evident in the literature [246,247,248,249,250,251]. Early life feeding practices, such as breastfeeding exclusivity, breastfeeding duration and the introduction of supplemental infant formula are likely influenced by non-clinical factors such as parental choice, and inevitably, the psychological and emotional wellbeing of the mother, the infant–mother dyad, and their support networks, although robust CF-specific evidence on maternal feeding experiences is limited [182,185]. Prospective, longitudinal qualitative or mixed methods studies which seek to explore parental attitudes and experiences throughout the infant feeding period are crucial to our holistic understanding of early life feeding practices and will assist in guiding targeted interventions for the optimization of infant feeding in the first 1000 days of life.

### 5.6. CFTR Modulator Therapies and Early Life Feeding

The potential for therapeutic modulation of CFTR dysfunction in the first 1000 days of life offers the very real possibility that early life feeding goals might parallel that of children without CF in the future. However, to date, there is no evidence on the impact of CFTRm therapy on breastfeeding, complementary feeding practices or dietary intake within the first 1000 days of life. It has been acknowledged in the literature that clinical trials of CFTRm therapy in infancy are challenging due to the vulnerability of this age group, potential for harm and a lack of well-established outcome measures [252]. It may be that early life feeding outcomes, such as breastfeeding duration, may strengthen the pool of relevant and meaningful outcome measures utilized in this unique age group and provide the evidence for the impact of CFTRm therapies on early life nutritional optimization.

## 6. Future Considerations: Early Life Feeding to Promote Healthy Aging in Cystic Fibrosis

This review has highlighted the substantial impact of early life CF disease on physical growth and optimal nutrition in cwCF during the first 1000 days of life. MI, EPI and other gastrointestinal manifestations influence a child’s growth trajectory and their exposure to exclusive and sustained breastmilk. In turn, growth trajectories within the first 1000 days influence lung health and disease progression. Early aggressive nutritional intervention, whilst critical to prevent undernutrition and any deleterious effects on lung health, may inadvertently contribute to rapid weight gain, beyond normative values or over and above a child’s genetic potential, and we do not yet understand what impact rapid weight gain in cwCF in the first 1000 days of life has on future cardiometabolic disease. Further research alongside considered thought and discussion as to what represents ‘healthy growth’ in the first 1000 days of life for a cwCF is warranted. The balance between sufficient early growth to optimize pulmonary and developmental outcomes, potentially at the expense of exclusive breastfeeding, with the hypothetical long-term effects of excessive catch-up growth on later life metabolic complications, represents a unique challenge in CF.

Optimal nutrition in the first 1000 days of life for cwCF is somewhat of a paradox. Breastfeeding rates continue to fall well short of those seen in typically developing children and yet there are amplified benefits, known and presumed, of the bioactive components of breastmilk on lung health, bacterial proliferation, inflammatory pathways and the microbiome. The barriers to breastfeeding exclusivity and duration in cwCF are thought to be numerous, encompassing disease-related factors, socio-environmental factors and maternal psychological and emotional wellbeing, although our knowledge in this area is still developing. Qualitative research exploring deeper insights into the barriers and enablers to successful breastfeeding in CF, both from a clinician perspective and caregiver perspective, is warranted. Supportive interventions designed to extend the duration of ‘any’ breastmilk, regardless of exclusivity, is warranted and may be particularly beneficial in children with early life lung disease. Finally, the impact of breastfeeding cwCF on systems beyond the lungs, including markers of inflammation and the microbiome, is a crucial area of research and will provide a key pillar of knowledge for driving advocacy and supportive interventions within CF centers aimed at increasing breastfeeding duration in this vulnerable population.

**Figure 1:**
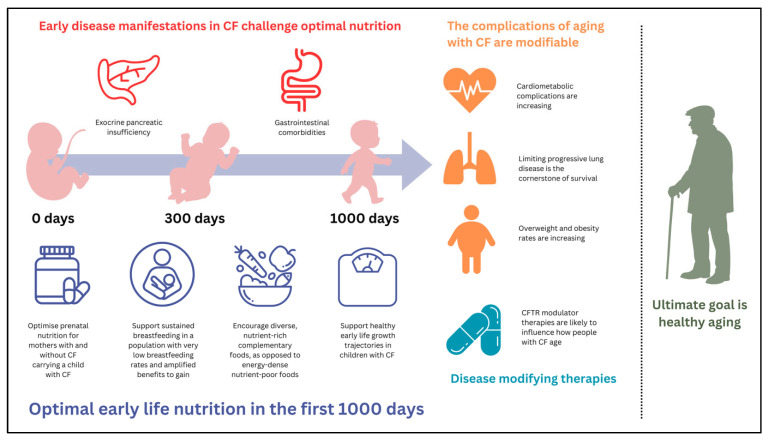
Early life nutrition: the first 1000 days and healthy aging in cystic fibrosis.

Similarly, diet quality in cwCF is suboptimal and yet the benefits of nutrient-dense foods for a condition which is characterized by poor nutrient absorption, reduced levels of circulating EFAs and dysbiosis are thought to be immense. The long-term benefits of a high-quality diet, rich in diversity, on outcomes such as chronic disease, obesity, inflammatory pathways, metabolic health, gut microbiome, cognitive function and longevity are important outcomes to consider in an aging CF population. Awareness amongst researchers and clinicians on the importance of diet quality in CF is increasing and, therefore, the continued monitoring of dietary balance in cwCF, including complementary ‘first’ foods, will be important in the future to ensure translation and uptake of evidence-based practice. Figure 1 provides a pictorial summary of the positive and negative influences on early life growth and nutrition in the first 1000 days of life, as summarized in this review, and how these may be hypothetically associated with lifelong healthy aging in pwCF.

## 7. Limitations

Due to several well-known concepts of nutritional programming being applied to the CF context, some key limitations must be highlighted. The first is that the ‘first 1000 days’ and ‘DOHaD’ concepts are heavily grounded on evidence from LMICs where malnutrition, poverty and unsafe and unsanitary environmental conditions are common. CwCF, particularly those living in high income countries, are exposed to developmental influences that are not only socio-economically different but physiologically and metabolically unique. Applying these constructs to the CF population is hypothetical and intended to generate questions in a rapidly evolving era of CF healthcare where the future goal should be to achieve optimal early life feeding, nutrition and growth outcomes in keeping with those of children without CF.

The second key limitation is that the evidence presented in this review on early life nutrition, growth and feeding is from a largely CFTRm naïve population which may not be directly transferable to the emerging CF population who are likely to be treated with disease-modifying drugs within the first two years of life. However, it is unlikely that infants under three-four months of age will be exposed to CFTRm on a large scale in the near future, and, therefore, understanding the barriers and facilitators to breastfeeding exclusivity and duration, alongside interventions to optimize breastfeeding rates in this vulnerable population will continue to be of relevance. Additionally, there is a stark absence of robust longitudinal studies in infants and young children treated with CFTRm therapies where nutrition and feeding outcomes are the primary endpoints.

Finally, a topic not within the scope of this review, but one that is equally important, are the socioeconomic disparities that exist globally in CF healthcare, raising the issue of applicability and generalizability of evidence presented within this review. With that being said, championing quality prenatal nutrition, breastfeeding duration, and nutrient-dense complementary foods, are not pillars of early life nutrition which should only apply to pwCF living in well-resourced settings and high-income countries. Indeed, these three pillars of early life nutrition are the bones of the ‘first 1000 days’ nutritional framework intended to support optimal development for children living in social, economic and environmental disadvantage.

## 8. Conclusions

The first 1000 days represent a critical window to optimize growth and nutrition in cwCF. Three priorities emerge from this review. First, we must establish evidence-based definitions of healthy growth trajectories specific to children living in the modern era of CF, considering not only optimization of pulmonary health but potential influences of early growth patterns on later life cardiometabolic outcomes. Second, sustained advocacy is needed to champion breastfeeding in cwCF, where clinically and psychologically appropriate, to at least match those of children without CF, recognizing breast milk as an underutilized nutritional advantage. Third, we must lead the way in investigating dietary diversity and quality in medically complex children—a research gap with far-reaching implications for both immediate outcomes and long-term healthy aging. Early life CFTRm therapies now offer a timely opportunity to fundamentally reshape nutritional management and outcomes in cwCF. Addressing these priorities will help to establish a stronger foundation for lifelong healthy aging in CF.

## Data Availability

No new data were created or analyzed in this study. Data sharing is not applicable to this article.
